# Increased risk of non-AIDS-defining cancers in Asian HIV-infected patients: a long-term cohort study

**DOI:** 10.1186/s12885-018-4963-8

**Published:** 2018-11-06

**Authors:** Naoyoshi Nagata, Takeshi Nishijima, Ryota Niikura, Tetsuji Yokoyama, Yumi Matsushita, Koji Watanabe, Katsuji Teruya, Yoshimi Kikuchi, Junichi Akiyama, Mikio Yanase, Naomi Uemura, Shinichi Oka, Hiroyuki Gatanaga

**Affiliations:** 1 Departments of Gastroenterology and Hepatology, National Center for Global Health and Medicine, 1-21-1 Toyama, Shinjuku-ku, Tokyo, 162-8655 Japan; 2 AIDS Clinical Center, National Center for Global Health and Medicine, 1-21-1 Toyama, Shinjuku-ku, Tokyo, 162-8655 Japan; 3 Department of Gastroenterology, Graduate School of Medicine, the University of Tokyo, Bunkyo-ku, Tokyo 113-8655 Japan; 4 Department Director, Department of Health Promotion, National Institute of Public Health, 2-3-6 Minami, Wako, Saitama, 351-0197 Japan; 5 Department of Clinical Research, National Center for Global Health and Medicine, 1-21-1 Toyama, Shinjuku-ku, Tokyo, 162-8655 Japan; 6 Department of Gastroenterology and Hepatology, Kohnodai Hospital, National Center for Global Health and Medicine, 1-7-1, Kohnodai, Ichikawa, Chiba, 272-8516 Japan

**Keywords:** Non-AIDS-defining malignancies, Gastric cancer, Colorectal cancer, Liver cancer, Lung cancer, All-cause mortality, Hepatitis viral infection, Highly active antiretroviral therapy

## Abstract

**Background:**

Data on the long-term risks of non-AIDS defining cancers (NADCs) are limited, especially in Asians. The incidence of NADCs may correlate with the epidemiological trend of cancers or oncogenic infection in each country, and thus the target cancers would be different between Western and Asian countries. We aimed to elucidate the incidence of NADCs and its predictive factors in Asian HIV-infected patients.

**Methods:**

Subjects were HIV-infected patients (*n* = 1001) periodically followed-up for 9 years on average. NADCs were diagnosed by histopathology and/ or imaging findings. Standardized incidence ratios (SIR) were calculated as the ratio of the observed to expected number of NADCs for comparison with an age-and sex-matched general population. Cox’s proportional hazards model was used to estimate hazard ratios (HR).

**Results:**

During the median follow-up of 9 years, the 10-year cumulative incidence of NADCs was 6.4%.At NADC diagnosis, half of patients presented at age 40–59 years and with advanced tumor stage. Compared with the age-and sex-matched general population, HIV-infected patients are at increased risk for liver cancer (SIR, 4.7), colon cancer (SIR, 2.1), and stomach cancer (SIR, 1.8). In multivariate analysis, a predictive model for NADCs was developed that included age group (40–49, 50–59, 60–69, and ≥ 70 years), smoker, HIV infection through blood transmission, and injection drug use (IDU), and HBV co-infection. The c-statistic for the NADCs predictive model was 0.8 (95%CI, 0.8–0.9, *P* < 0.001). The higher 10-year incidence rate of NADCs was associated with increasing prediction score.

**Conclusions:**

Liver and colon cancer risk was elevated in Asian HIV-infected individuals, similar to in Western populations, whereas stomach cancer risk was characteristically elevated in Asian populations. Half of Asian NADC patients were aged 40–59 years and had advanced-stage disease at diagnosis. Periodic cancer screening may be warranted for high-risk subpopulations with smoking habit, HIV infection through blood transmission or IDU, and HBV co-infection, and screening should be started over 40 years of age.

## Background

The incidence of non-AIDS-defining cancers (NADCs) has been increasing and is a significant source of morbidity and mortality in the HIV-infected population [1–7]. Most data on NADC risk are available for Western countries [1–3, 6, 7] and little data from Asia are available [4, 5]. Compared with the general population, people infected with HIV have an increased risk of some NADCs [1–7], and NADCs are now being diagnosed at a much younger age [8, 9]. However, there are no definitive guidelines for NADC screening, and most organization guidelines for cancer screening do not make recommendations that are different for HIV-infected patients. As it stands, most clinicians caring for HIV patients may perform only age-appropriate cancer screening [10].

NADC screening should be effective and low cost, and it is therefore recommended for people who are at high risk for cancer*.* However, few studies have investigated the detailed risk factors for NADCs. In addition, it is not yet known which cancers should be noted in screening in the long-term management of HIV-infected patients. It is possible that the incidence of NADCs may correlate with the epidemiological trend of cancers or oncogenic infection in each country, and thus the target cancers would be different between Western and Asian countries.

In consideration of these issues, we aimed to elucidate i) the incidence of NADCs during the long-term follow-up of HIV patients, ii) the standardized incidence ratio (SIR) of each NADC in comparison with the general Japanese population, and iii) predictive factors for NADC development.

## Methods

### Study design, setting, and participants

We conducted a retrospective cohort study using data from a prospectively recorded electronic database (MegaOak online imaging system, NEC, Japan, and SolemioEndo, Olympus, Japan) between January 1997 through December 2015. This database is a searchable collection of records into which physicians and nurses immediately input all clinical findings after patient examinations. We included 1185 Japanese adult patients with HIV type 1 (HIV-1) infection who were diagnosed at the AIDS Clinical Center and/or Department of Gastroenterology and Hepatology, or were referred from other medical institutions to the AIDS Clinical Center at the National Center for Global Health and Medicine, Tokyo, Japan. More than 95% of patients initially visited the AIDS Clinical Center. All HIV-infected patients were followed-up at the AIDS Clinical Center and were managed by HIV infection experts. Our hospital has one of the largest HIV clinics, treating approximately 15% of HIV-infected patients in Japan [11]. First, we reviewed all radiologic, endoscopic, surgical, pathological, and other clinical data in the electronic medical record system for these 1185 patients. We excluded the following patients: i) those referred from other hospitals for treatment or investigation for cancer and those who had cancer before or at the time of diagnosis of HIV infection at our institution (*n* = 28); (ii) those whose clinical information could not be sufficiently collected (*n* = 31); iii) those who visited our hospital only once (*n* = 176); and iv) those whose HIV infection was diagnosed 0–3 months prior to review (*n* = 41) because the cancer risk in this period can appear artificially high due to intensive medical evaluations. More than one exclusion criterion applied to some patients. Finally, a cohort of 1001 HIV-infected patients who were periodically followed up was selected for analysis. Because this was a retrospective cohort study that was conducted without invasive procedures, and patient information was anonymized and deidentified before analysis, the requirement for patient consent was waived. This study was approved by the ethics committee of the National Center for Global Health and Medicine (Nos.1424 and 1440) and was implemented in accordance with the provisions of the Declaration of Helsinki.

### Data collection and follow-up

In accordance with our specific institutional protocol, all patients when first seen at our hospital were systematically screened for hepatitis B virus (HBV) and hepatitis C virus (HCV) infection and their CD4 cell count and HIV-1 viral load were determined [12]. A positive result for the hepatitis B surface antigen indicated HBV infection. In Japan, because universal vaccination against HBV has not been introduced and intervention to prevent mother-to-child transmission has been highly successful [13], most adult cases with chronic HBV infection are considered to be sexually transmitted [14]. HCV infection was defined as the presence of HCV antibodies. Since 1997, patients on their first visit to the AIDS clinical center have completed a detailed, structured questionnaire during a face-to-face interview with well-trained researchers in a private room. Patients were asked about their lifestyle habits (smoking history and alcohol consumption), medications, past history and co-morbidities, and presumed route of transmission for HIV infection (men who have sex with men [MSM], heterosexual, blood transmission, or injection drug use [IDU]). During the follow-up period, most patients visited our hospital every 1–3 months. The prescription period for all drugs under the health care system is limited to 3 months in Japan, so patients need to make visits at least once during this period for prescriptions as well as for monitoring of CD4 cell count, HIV viral load, or serological tests. In Japan where there is a high incidence of gastric, colorectal, liver, and lung cancers, endoscopy and computed tomography (CT) are frequently performed for cancer screening, even as part of routine examinations for patients without symptoms of the disease.

### Diagnosis of non-AIDS-defining cancers

The diagnosis of NADCs was based on histopathological or cytological examination of the resected specimen, biopsy, tumor brush, or fluid samples obtained percutaneously, during upper or lower endoscopy, bronchoscopy, or surgery. When a cancer was suspected or confirmed, MDCT with other imaging modalities was performed in all patients. Gastrointestinal cancer included cancers affecting the oral cavity, esophagus, stomach, colon, and anorectum. Staging of NADCs was based on the TNM classification, and tumors were staged according to the Union for International Cancer Control (UICC) classification [15].

### Statistical analysis

To estimate the actual incidence of the respective NADCs, we analyzed those patients who did not have NADC before and at the time of diagnosis of HIV infection at our institution (index date). We followed up patients from the diagnosis of HIV infection at our institution (index date) to the diagnosis of any NADC as the primary endpoint. Data were censored at the time of the last visit, or death, or at the end of the follow-up period (December 31, 2015). The diagnosis of NADCs was defined as occurrence of first primary cancer, and multiple cancer per patient was not assessed in this study. The Kaplan–Meier method was used to estimate the cumulative incidence of any NADC at 1, 5, 10, and 15 years.

In the comparison with the general population, we evaluated the expected cancer rate based on vital statistics data for Japan. The expected number of NADCs was determined using age-stratified and sex-specific data on the incidence of cancer in Japan, provided by the Center for Cancer Control and Information Services, National Cancer Center, Japan [16]. Because cancer rates are provided for 5-year age groups in Japan, 5-year age groups were used for the calculation of age-specific rates, for the cumulative rate 20–84 years of age. The standardized incidence ratio (SIR) was calculated as the ratio of the observed number to the expected number of patients developing NADCs. The 95% confidence interval (CI) of the SIR was estimated, assuming a Poisson distribution following a variance-stabilizing transformation. Statistical analysis of SIR was performed using SAS software version 9.4 (SAS Institute, Cary, NC, USA).

Predictive factors were assessed at enrollment, and time-fixed covariates were included in the time-to-event models. Predictive factors for NADCs were examined using Cox’s proportional hazards models. We estimated unadjusted and adjusted hazard ratios (HRs) and 95% confidence intervals (CIs). In multivariate analysis, we adjusted for factors including age category, smoker, HIV risk category, HBV infection, HCV infection, diabetes mellitus with end-organ damage, congestive heart failure, and chronic moderate to severe liver disease that showed an association with NADC (*p* < 0.2) in univariate analysis. A final model was then developed by backward selection of factors showing values of *p* < 0.1. The weight of each predictor was determined according to the coefficients in the model. Based on the final model’s regression coefficients, points were assigned as follows: 1 point each for blood transmission and HBV infection; 2 points each for age 40–49, smoker, and injection drug use; 3 points each for age 50–59 and age 60–69; and 4 points for age ≥ 70. The accuracy of the predictive model for NADCs was evaluated by the c-statistics using Harrell’s method. [17] Values of *p* < 0.05 were considered significant. All statistical analyses were performed using STATA version 13 software (StataCorp, College Station, TX).

## Results

### Baseline characteristics

Patient characteristics are shown in Table 1. Various HIV risk groups were identified: the majority of patients were MSM at 75.2%, followed by heterosexuals at 12.3%, hemophilia at 9.5%, IDU at 0.8%, and transfusion at 0.5%. At enrollment, median CD4 was 100 cells/μL and median HIV-VL was 63,000 copies/mL. The proportion of patients receiving antiretroviral therapy at enrollment was 31.2%. The positive rates of HBV, HCV, and both HBV and HCV infection in our cohort were 15.1%, 14.5%, and 1.5%, respectively. Among the comorbidities, the most common were chronic liver disease (14.1%), dyslipidemia (12.9%), hypertension (10.2%), diabetes mellitus (8.0%), and chronic kidney disease (6.4%) in this order.

**Table 1:** Patients characteristics (*N* = 1001)

Median age (IQR), years	38 (31–48)
20–39	551 (55.0%)
40–49	239 (23.9%)
50–59	139 (13.9%)
60–69	56 (5.6%)
≥ 70	17 (1.7%)
Sex (male)	942 (94.1%)
Alcohol drinker	557 (55.6%)
Smoker	421 (42.1%)
Year of entry	
1997–2001	267 (26.7)
2002–2006	331 (33.1)
2007–2011	309 (30.9)
2012–2015	94 (9.4)
HIV-related factors	
HIV risk group	
MSM	753 (75.2%)
Heterosexuals	123 (12.3%)
Blood transmission^a^	99 (9.9%)
Injection drug use	8 (0.8%)
Unknown	18 (1.8%)
Median CD4 (IQR), cells/μL	100 (30–184)
CD4 < 200, cells/μL	777 (77.6)
200–499	209 (20.9)
≥ 500	15 (1.5)
Median HIV VL (IQR), copies/mL	63,000 (5400-310,000) (4.4–5.6)
HIV VL < 400, copies/mL	166 (16.6)
400–75,000	360 (36.0)
> 75,000	475 (47.5)
On antiretroviral therapy	312 (31.2)
HBV infection alone	151 (15.1%)
HCV infection	145 (14.5%)
HBV and HCV co-infection	15 (1.5%)
Co-morbidities	
Hypertension	102 (10.2%)
Dyslipidemia	129 (12.9%)
Diabetes mellitus (uncomplicated)	80 (8.0%)
Diabetes mellitus (end-organ damage)	32 (3.2%)
Myocardial infarction	9 (0.9%)
Congestive heart failure	15 (1.5%)
Peripheral vascular disease	2 (0.2%)
Cerebrovascular disease	16 (1.6%)
Dementia	14 (1.4%)
COPD	7 (0.7%)
Connective tissue disease	2 (0.2%)
Peptic ulcer disease	31 (3.1%)
Chronic kidney disease	64 (6.4%)
Hemiplegia	1 (0.1)
Chronic liver disease	141 (14.1%)
Mild	105 (10.5%)
Moderate to severe	36 (3.6%)

Note. ^a^A total of 96% had hemophilia

Abbreviations: *HBV* hepatitis B virus, *HCV* hepatitis C virus, *HAART* highly active anti-retroviral therapy, *IQR* interquartile range, *MSM* men who have sex with men, *IDU* injection drug users, *VL* viral load, *COPD* chronic obstructive pulmonary disease, *NADCs* non-AIDS-defining cancers

### Development of NADCs and comparison with the general population

**Fig. 1:**
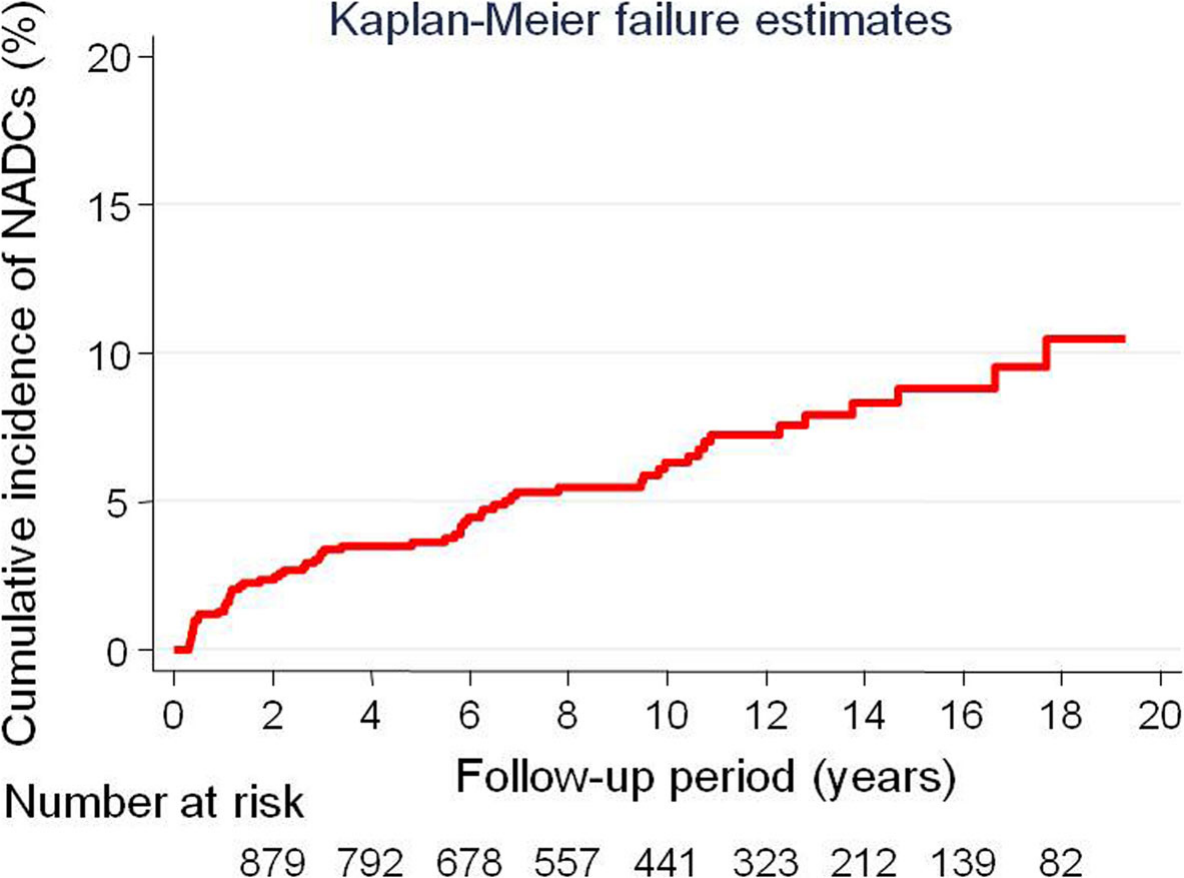
The cumulative incidence of developing NADCs.The cumulative probability of NADCs (95% confidence interval) at 1, 5, 10, and 15 years was 1.3% (0.77–2.3), 3.7% (2.6–5.1), 6.4% (4.8–8.3), and 8.8% (6.7–11.6), respectively

During a median follow-up of 9.0 years, 61 NADCs (6.1%) developed in 1001 HIV patients (Table 2). The overall incidence of NADCs was 9.19 per 1000 person-years. Total follow-up time was 8756.7 person-years. The cumulative probability of NADCs at 1, 5, 10, and 15 years was 1.3%, 3.7%, 6.4%, and 8.8%, respectively (Fig. 1). The characteristics of patients at the time of NADC diagnosis are shown in Table 2. Half of patients (49.2%) had an advanced tumor stage (III or IV) according to the Union for International Cancer Control classification. Median age was 57 years. The majority of HIV patients diagnosed with NADCs were in the age group 40–59 years at 50.8%, followed by the patients in the age group 60–79 years at 37.7%. All patients received antiretroviral therapy at the time of NADC diagnosis.

**Table 2:** Development of non-AIDS-defining cancers (NADCs) in patients with HIV infection (*N* = 1001)

Development of NADCs	61 (6.1%)
Development of gastrointestinal NADCs	31 (3.1%)
Median follow-up period (IQR), years	9.0 (4.8–13.2)
Stage at the time of NADC diagnosis^a^	
0 or I	14 (22.9%)
II	17 (27.9%)
III	9 (14.8%)
IV	21 (34.4%)
Age at the time of NADC diagnosis^a^, years	
Median age (IQR)	57 (49–65)
20–39	5 (8.2%)
40–59	31 (50.8%)
60–79	23 (37.7%)
80–89	2 (3.3%)
Antiretroviral therapy at the time of NADC diagnosis^a^	61 (100%)

Note. ^a^Number and percentages were calculated among patients with NADCs (*n* = 61). Gastrointestinal cancer included cancers affecting the oral cavity, esophagus, stomach, colon, and anorectum.Abbreviations: *IQR* interquartile range, *NADCs* non-AIDS-defining cancers

The observed number and SIR of each NADC are listed in Table 3. Half of NADCs were gastrointestinal cancer. Among NADCs, the incidence of liver cancer, colon cancer, and gastric cancer was significantly increased in HIV patients relative to the general population. Their SIR was as follows: liver cancer, 4.7; colon cancer, 2.1; and gastric cancer, 1.8.

**Table 3:** Observed and expected number of non-AIDS-defining cancers (NADCs) and all-cause deaths in patients with HIV compared with the general population in Japan

Cancer type	Observed	Expected	SIR (95% CI)
Liver	9	1.9	4.7 (2.1–8.2)
Colorectal	15	7.7	1.9 (1.1–3.0)
Colon	10	4.7	2.1 (1.0–3.7)
Anorectal*	5	3.1	1.6 (0.5–3.4)
Gastric	11	6.0	1.8 (1.0–3.1)
Lung	8	4.4	1.8 (0.8–3.3)
Oral cavity and pharynx	3	1.0	2.9 (0.5–7.0)
Esophageal	2	1.4	1.4 (0.1–4.0)
Pancreas	1	1.2	0.8 (< 0.1–3.3)
Biliary tract	2	0.6	3.2 (0.3–9.2)
Bladder	2	0.8	2.4 (0.2–6.7)
Skin	1	0.6	1.8 (< 0.1–6.9)
Prostate	1	4.0	0.2 (< 0.1–1.0)
Thyroid	1	0.6	1.7 (< 0.1–6.5)
Breast	1	0.8	1.3 (< 0.1–5.0)
Hodgkin lymphoma	4	NA^a^	NA^a^

Note. *Anorectal cancer included rectal cancer (*n* = 2) and anal cancer (*n* = 3). ^a^General population data were not available for Hodgkin lymphoma

Abbreviation Standardized incidence ratio, *SIR* standardized mortality ratio

### Predictive factors for NADCs

The predictive factors for NADCs are shown in Table 4. In the univariate analysis, factors associated with NADC (*p* < 0.2) were advanced age, smoker, heterosexuality, IDU, HBV infection, HCV infection, diabetes mellitus (end-organ damage), congestive heart failure, and moderate to severe chronic liver disease. In multivariate analysis, a final prediction model for NADCs was developed that included age at 40–49, age at 50–59, age at 60–69, age ≥ 70, smoker, blood transmission, IDU, and HBV infection (Table 4). A predictive model for NADCs was developed using eight factors. Based on the final model’s regression coefficients (Table 4), points were assigned as follows: 1 point each for blood transmission and HBV infection; 2 points each for age 40–49, smoker, and injection drug use; 3 points each for age 50–59 and age 60–69; and 4 points for age ≥ 70. The c-statistic for the NADC predictive model was 0.8 (95%CI, 0.8–0.9, *P* < 0.001) (Table 5). The higher 5- and 10-year incidence rates of NADCs were associated with increasing prediction score. The HRs for NADCs were also associated with increasing prediction score (*p* < 0.001 for trend).

**Table 4:** Risk factors for non-AIDS-defining cancers (NADCs) in HIV-infected patients (*N* = 1001)

Factors	Crude HR (95% CI)	*P* value	Adjusted HR^a^ (95% CI)	Coefficient^a^ (95% CI)	*P* value
Age < 40 (years)	1 (reference)		1 (reference)		
40–49	5.1 (2.2–11.6)	< 0.001	5.4 (2.4–12.3)	1.7 (0.9–2.5)	< 0.001
50–59	12.7 (5.8–27.7)	< 0.001	15.3 (6.7–34.6)	2.7 (1.9–3.5)	< 0.001
60–69	13.0 (5.0–34.1)	< 0.001	17.1 (6.4–46.2)	2.8 (1.9–3.8)	< 0.001
≥ 70	36.4 (12.8–103.7)	< 0.001	47.3 (16.2–138.1)	3.9 (2.8–4.9)	< 0.001
Sex (male)	1.3 (0.4–4.2)	0.653			
Alcohol drinker	1.0 (0.6–1.7)	0.876			
Smoker	4.5 (2.5–8.1)	< 0.001	5.4 (3.0–9.8)	1.7 (1.1–2.3)	< 0.001
HIV risk group, MSM or unknown	1 (reference)		1 (reference)	1 (reference)	
Heterosexuals	1.9 (1.0–3.7)	0.045	1.6 (0.8–3.2)	0.5 (−0.2–1.2)	0.142
Blood transmission	1.1 (0.5–2.4)	0.765	2.6 (1.2–5.8)	1.0 (0.1–1.8)	0.021
Injection drug use	9.1 (2.2–37.7)	0.002	5.4 (1.2–23.3)	1.7 (0.2–3.2)	0.025
CD4 < 200 (cells/μL)	0.7 (0.4–1.2)	0.222			
HIV VL > 75,000 (log_10_ copies/mL)	1.00 (0.6–1.6)	0.972			
HBV infection	1.8 (1.0–3.3)	0.059	1.8 (1.0–3.5)	0.6 (−0.03–1.2)	0.060
HCV infection	1.6 (0.9–2.9)	0.120			
Hypertension	1.2 (0.6–2.5)	0.619			
Dyslipidemia	0.8 (0.3–1.7)	0.494			
Diabetes mellitus (uncomplicated)	1.5 (0.7–3.2)	0.337			
Diabetes mellitus (end-organ damage)	2.3 (0.8–6.3)	0.111			
Myocardial infarction	1.8 (0.2–12.8)	0.569			
Congestive heart failure	2.6 (0.6–10.7)	0.182			
Peripheral vascular disease	NA*	NA*			
Cerebrovascular disease	1.9 (0.5–7.8)	0.373			
Dementia	1.2 (0.2–8.7)	0.857			
COPD	NA*	NA*			
Connective tissue disease	NA*	NA*			
Peptic ulcer disease	1.1 (0.3–4.4)	0.921			
Chronic kidney disease	0.5 (0.2–2.1)	0.352			
Hemiplegia	NA	NA*			
Chronic liver disease (mild)	1.2 (0.6–2.5)	0.680			
Chronic liver disease (moderate to severe)	2.6 (1.1–6.6)	0.037			

Note. *Statistical analysis could not be performed because there were no cases with the factor among patients with NADCs or those who died. ^a^Multiple Cox’s proportional hazard modeling was used with backward elimination for factors that were found to be significant (*p* < 0.2) on univariate analysis. Abbreviations: *CI* confidence interval, *HBV* hepatitis B virus, *HCV* hepatitis C virus, *MSM* men who have sex with men, *VL* viral load, *COPD* chronic obstructive pulmonary disease, *NADCs* non-AIDS-defining cancers

**Table 5:** Scoring model for the prediction of non-AIDS-defining cancers (NADCs) in HIV-infected patients (*N* = 1001)

Score	No. of NADCs/ Non-NADCs	Cumulative incidence rate of NADCs at 5 years (95% CI)	Cumulative incidence rate of NADCs at 10 years (95% CI)	Hazard ratio (95% CI)
0	2/ 222	0.9 (0.2–3.7)	0.9 (0.2–3.7)	1 (reference)
1	1/ 91	0	1.4 (0.2–9.7)	0.9 (0.08–10.2)
2	6/ 273	0.4 (0.1–2.8)	1.4 (0.4–4.4)	2.5 (0.5–12.3)
3	8/ 174	2.9 (1.2–6.9)	2.9 (1.2–6.9)	5.1 (1.1–23.9)
4	11/ 101	5.8 (2.7–12.5)	10.7 (5.5–20.4)	12.6 (2.8–56.7)
≥5	33/ 79	20.2 (13.5–29.7)	41.3 (29.7–55.3)	53.2 (12.7–222.5)
Total	61/ 940			P for trend < 0.001

C-statistics: 0.8 (95%CI, 0.8–0.9, *P* < 0.001)

Note. Based on the final model’s regression coefficients (Table 4), 1 point each was assigned to blood transmission, and HBV, 2 points each were assigned to age 40–49, smoker, and IDU, 3 points was assigned to age 50–59, age 60–69, and 4 points was assigned to age ≥ 70. Abbreviations: *CI* confidence interval, *HBV* hepatitis B virus, *IDU* injection drug use, *NADCs* non-AIDS-defining cancers

## Discussion

This long-term follow-up study focused on the development of NADCs and its predictive factors among 1001 HIV patients. Several important findings were noted. First, during the median follow-up of 9 years, the 10-year cumulative incidence of NADCs was 6.4%. Second, half of NADC patients were aged 40–59 years and had advanced-stage disease at diagnosis. Third, compared with the age-and sex-matched general population, HIV patients were at increased risk of liver, colon, and stomach cancers. Fourth, a predictive model for NADCs was developed that included age group (40–49, 50–59, 60–69, and ≥ 70 years), smoker, HIV infection through blood transmission, and injection drug use (IDU), and HBV co-infection.

To our knowledge*,* this is the first study to show a higher incidence of stomach cancer in HIV patients than in the general population. One study showed an elevated risk of stomach cancer in HIV patients compared with the general population, but the report has subsequently been retracted and the corrected SIR is no longer significantly elevated [18]. The high incidence of stomach cancer in Japan compared with Western countries is due to the higher prevalence of *Helicobacter pylori* infection [19], or the higher occurrence in Japan of the precancerous condition of intestinal metaplasia due to *H. pylori* infection compared with Western and other Asian countries [20, 21]. We suggest that decreased immunity in HIV patients may result in increased exposure to infection and severe gastritis, leading to increased risk of stomach cancer, because immune function is necessary to sustain *H. pylori* infection and the accompanying inflammation [22]. Although few studies have investigated the incidence of NADCs in Asia [4, 5], our result of a high incidence of liver and colon cancers in HIV patients compared with the general population is consistent with the results of reports from Western countries [1, 3, 6, 7]. The repartition of observed NADCs might be correlated with the cancers found commonly in each country. Indeed, the prevalent pattern of cancers in HIV patients in our study reflects the cancer incidence in Japan [16].

Our prediction scoring system could answer the question about which HIV patients should be screened for NADCs. In this study, the higher age groups of 40–49, 50–59, 60–69, and ≥ 70 with reference to age < 40 were associated with a greater hazard ratio for NADCs, in agreement with past reports [1, 23]. In addition, more than half of NADCs were diagnosed in the 40–59 age group. Because many people living with HIV can expect to live as long as those without HIV [24], the factor of age seems to be one of the most important to consider in cancer screening, and HIV patients should start receiving screening over 40 years of age.

In agreement with past reports [7, 25], smoking was an independent risk factor in our study. Smoking is more common among HIV patients, at 57%, than in those without, at 33% [25]. Smoking is also known to suppress the immune system [26] and increase cancer risk. A literature review showed many types of malignancies have been reported in persons with hemophilia irrespective of infection with HIV and they have high rates of malignancies compared with the general population [27]. Due to the high prevalence of oncogenic virus transmitted through blood [27, 28], HIV-infected hemophilia patients may have an increased risk of cancer. The reason for the unexpected finding of IDU was cancer risk; it is possible that heavy alcohol consumption or HCV infection is common among injection drug users [29, 30], which would affect the risk of NADCs.

In this study, half of NADC patients had advanced-stage cancer. Previous reports have shown that patients with NADCs often present with more advanced-stage disease and have a poor prognosis. For example, in a study of 41 HIV patients with hepatocellular carcinoma (HCC) and 384 controls with HCC, the HIV-infected patients had a higher risk of advanced HCC stage with multifocal and infiltrating lesions, and higher local recurrence and mortality [9]. These findings suggest that early screening and treatment of NADCs may improve the population’s poorer outcome, with rapid progression and high mortality.

Our study has some limitations. Although most of the patients visited our clinic regularly every 3 months as the maximum prescription period for any drug under the Japanese health care system is limited to 3 months, cancer screening strategies and its timing were at the discretion of the treating physicians. Second, this is not a population-based but a single-center study, so selection bias is not avoidable. For instance, due to high incidence of gastrointestinal cancer in Japan, HIV expert physicians may be likely to perform abdominal CT or endoscopy than those in Western countries, leading to the relatively higher incidence of gastrointestinal cancer. Third, we did not have any information of HBV-DNA or HCV-RNA level that may affect NADC risk. Because some factors such as CD4 or HIV-RNA levels can change during long-term follow-up, time-varying covariates may have been preferable to the time-to-event models, but time-varying covariates were not available. Fourth, the accuracy of the predictive model was relatively high (c-statistic, 0.8), but the model could lead to an overly optimistic evaluation. Because we could collect only a small sample of NADCs (*n* = 61) in our single-center study, a validation study with another population or at institutions with larger numbers of subjects is needed in the future. Finally, we have no data on cancer incidence in Tokyo and could not calculate the Tokyo-specific SIRs, so the expected numbers of events may be inappropriate if cancer incidence in Tokyo is different from the whole of Japan. Despite these limitations, there are several important strengths of this study. First, we could follow up > 75% of patients for > 5 years. Second, systematically screened laboratory tests and the interviews conducted at the first visit to our hospital enabled us to collect detailed risk factors including HIV-related factors, HBV, HCV, and comorbidities for all patients. Third, the diagnosis of cancer was based on histopathological or cytological examination with multiple imaging modalities, which could assess cancer staging.

In conclusion, liver and colon cancer risk was elevated in Asian HIV-infected individuals, similar to in Western populations, whereas stomach cancer risk was characteristically elevated in Asian populations. Half of NADC patients were aged 40–59 years and had advanced-stage disease at diagnosis. Periodic cancer screening may be warranted for high-risk subpopulations with smoking habit, HIV infection through blood transmission or IDU, and HBV co-infection, and screening should be started over 40 years of age.

## Abbreviations

CI: Confidence interval
COPD: Chronic obstructive pulmonary disease
HAART: Highly active anti-retroviral therapy
HBV: Hepatitis B virus
HCV: Hepatitis C virus; men who have sex with men
IDU: Injection drug users
IQR: Interquartile range
MSM: Men who have sex with men
NADCs: Non-AIDS-defining cancers
SIR: Standardized incidence ratio
SMR: Standardized mortality ratio
VL: Viral load
